# Paediatric upper limb trauma: what can we do to support a change in radiographic referral practice?

**DOI:** 10.1002/jmrs.633

**Published:** 2022-11-10

**Authors:** Maryann Hardy

**Affiliations:** ^1^ Diagnostic Radiography University of Bradford Bradford UK

## Abstract

Referral practices for upper limb trauma radiography in children vary. Knowledge of the influence of mechanism of injury and functional anatomy on trauma presentation can reduce unnecessary referrals for multiple concurrent radiographic examinations.

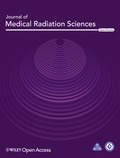

Children account for approximately 30–40% of emergency department attendances and the large majority of these relate to upper limb trauma.^1^, ^2^ Paediatric upper limb radiography, and the determination of normal from abnormal appearances as a consequence of anatomical growth and developmental changes, have long been a cause of concern for medical and non‐medical clinicians working in the acute trauma setting. With litigation associated with missed or misdiagnosed injuries increasing within the developed world, it is unsurprising that some centres dealing with paediatric patients are cautious, referring children for multiple, rather than focused, radiographic projections. This phenomenon, and the value it may have in identifying concomitant injuries, is considered in the paper “Is there benefit to concurrent X‐ray imaging of the wrist, forearm and elbow in paediatric patients following a fall on the outstretched hand?” by Sgualdino et al.^3^ within this issue. However, if we consider every radiographic projection to be a prescription for radiation, then any examination without clear justification for the exposure falls outside international radiation protection guidance and standards. So, what can we do to support a change in radiographic referral practice?

Firstly, it is important to understand the typical mechanisms of injury occurring in upper limb trauma in children and their clinical presentation. As soon as a child becomes mobile, they are at risk of falling. Once children have mastered mobility on the ground then their attention turns to climbing, increasing the risk of injury as a consequence of falling from a height, and then to wheeled activities (roller skates and skateboards) where a range of directional forces impacts the likelihood and type of injury. In all falls, the child will typically extend their arms to protect themselves. The impact with the ground creates a force that travels from the distal to the proximal end of the limb with the site of injury being dependent upon limb position at time of impact. Unfortunately, very few referrals provide specific details of the incident, direction of fall or limb position on impact and instead use the catchall acronym FOOSH (fall on out stretched hand) to convey the reason for radiographic imaging.

Next is to understand where injuries occur and their presentation. Forearm fractures, including the wrist, account for approximately two‐thirds of upper limb fractures in children of which over 60% occur at the distal radius and ulna (wrist). The mechanism for distal forearm injuries is typically FOOSH but forced dorsi‐ or volar flexion may have the same result.^4^ In older children, these injuries are often associated with point tenderness and swelling on clinical assessment but in toddlers, swelling may be difficult to detect and slight reddening may be the only sign of injury. The child will also typically present with the hand pronated and, in older children, supported by the unaffected arm with the elbow flexed. Where a fracture to the midshaft of the forearm is suspected then presentation may be different and an obvious deformity evident. Midshaft fractures typically occur at the junction between the middle and distal 1/3rd of forearm and the relative position of the attachments of the pronator and supinator muscles will often account for fracture fragment positions. In midshaft fractures, both the distal and proximal fracture fragments are in relative neutral positions with the distal fragment slightly pronated and proximal fragment slightly supinated. This can be seen by how the child holds the forearm on clinical examination. With fractures to the proximal radius and ulna, distal fragment pronation and proximal fragment supination is exaggerated and, as a consequence of the site of muscle attachment, the range of forearm motion will be increasingly restricted with increasing proximal fracture location. All these clinical presentation signs are useful in determining the likelihood of forearm injury and also the most appropriate imaging referral projection. Radiographers should expand their clinical assessment and presentation knowledge to assist in justifying radiographic referrals. Interestingly, in the paper above by Sgualdino et al.,^3^ three of the eight injuries outside the area of interest of the original imaging referral related to forearm fractures, all of which should potentially have been correctly identified by careful clinical assessment and understanding of functional anatomy and the impact of muscle action on forearm motion and presentation.

Considering the paediatric elbow, the most common fracture in the elbow region, accounting for 50–80% of presentations, is the supracondylar fracture. It typically occurs between 3 and 10 years of age with peak incidence between children 5 and 8 years.^5^ The child will typically present with some swelling around the elbow, holding the arm straight with the hand in pronation and may refuse to use the arm or flex the elbow.^6^ Like injuries to the wrist and forearm, the mechanism of injury is typically FOOSH with the elbow extended (95% of cases) resulting in the distal humeral fragment being displaced or angled posteriorly relative to the humeral shaft. The relatively uncommon flexion injuries are caused by a fall onto a flexed elbow and result in the distal humeral fragment being displaced or angled anteriorly relative to the humeral shaft.^4^ However, sometimes these injuries can be subtle, and therefore, increasing reliance has been placed on displacement of the elbow fat pads, associated with bleeding and oedema within the capsule, to support identification of the injury where radiographic evidence of fracture is unclear. As can be seen in the paper by Sgualdino et al.,^3^ four out of the eight concomitant injuries identified related to raised elbow fat pads, but is this really a positive finding? The appearance of fat pads is dependent on radiographic technique and degree of elbow flexion on imaging. There is also international debate on what defines a ‘raised’ fat pad, particularly in relation to the anterior fat pad, which was the concomitant abnormality identified in three of the cases in the paper by Sgualdino et al.^1^ Popellaars et al.^7^ determined that a raised anterior fat pad should be at an angle of at least 16° relative to the anterior humeral line but again, the accuracy of this measurement is dependent on the quality of radiographic technique. Importantly, the incidence of an occult fracture being present when a positive fat pad sign is observed has been estimated to be 45%^8^ and in a prospective study evaluating the presence of occult paediatric elbow injury using MRI,^9^ only 6/26 cases with a positive fat pad sign had an occult fracture, only one of which was a supracondylar fracture, the remaining five being fractures of the proximal radius or ulna.

So, considering all the evidence above, we can concur with the conclusions of Sgualdino et al.^3^ in questioning the appropriateness of referral for multiple concurrent, rather than focused, radiographic examinations of the upper limb in paediatric patients. We, as a radiography profession, must acknowledge how our ignorance of the impact of radiographic technique on diagnostic quality, and lack of engagement in patient assessment, has contributed to the practice of referring for multiple rather than focused radiographic examinations of the paediatric upper limb. The question going forward is how do we ensure that everyone involved in the patient pathway engages with the evidence base to facilitate change?

## Conflict of Interest

The author declares no conflict of interest.
